# Supplementary material for: Efficacy and acceptability of blue-wavelength light therapy for post-TBI behavioral symptoms: A systematic review and meta-analysis of randomized controlled trials
Source: PLoS One. 2022 Oct 6;17(10):e0274025. doi: 10.1371/journal.pone.0274025 (PMC9536631; doi:10.1371/journal.pone.0274025)

SUPPLEMENTARY MATERIALS

Efficacy and Acceptability of Blue-Wavelength Light Therapy for Common Behavioral Symptoms After Traumatic Brain Injury: A Systematic Review and Meta-Analysis of Randomized Controlled Trials

Karan Srisurapanont[1]; Yanisa Samakarna[1]; Boonyasit Kamklong[1]; Phichayakan Siratrairat[1]; Arina Bumiputra[1]; Montita Jaikwang1; Manit Srisurapanont, M.D.[2]*

1 Faculty of Medicine, Chiang Mai University, Chiang Mai, Thailand
2 Department of Psychiatry, Faculty of Medicine, Chiang Mai University, Chiang Mai, Thailand

Correspondence to:
Manit Srisurapanont, MD
Department of Psychiatry
Faculty of Medicine, Chiang Mai University
Muang, Chiang Mai 50200 THAILAND
Email: manit.s@cmu.ac.th

List of abbreviations:
BWLT = Blue-wavelength light therapy
CI = Confidence interval
IV = Inverse variance
LED = Light emitting diode
SMD = Standardized mean difference
TBI = Truamatic brain bnjury

CONTENTS

	Page
SUPPLEMENTARY TABLES	
S1 Table: Search Strategy and Results (April 13, 2022)	1
S2 Table: Excluded Trials with Reasons	5
SUPPLEMENTARY FIGURES (SFigures)	
S1 Fig.: Forest plots comparing the dropout rates	6
S2 Fig.: Subgroup analysis of five outcomes	7
S3 Fig.: Sensitivity analysis of five outcomes	9
S4 Fig.: Funnel plot showing the treatment-effect estimates of dropout rates	11

SUPPLEMENTARY TABLES

S1 Table: Search Strategies and Results (April 13, 2022)

A. PUBMED (n = 42)

Search number	Query	Search Details	Results
5	#1 AND #2 AND #3 AND #4	(“brain injuries”[MeSH Terms] OR (“brain”[All Fields] AND “injuries”[All Fields]) OR “brain injuries”[All Fields] OR (“brain”[All Fields] AND “injury”[All Fields]) OR “brain injury”[All Fields] OR (“craniocerebral trauma”[MeSH Terms] OR (“craniocerebral”[All Fields] AND “trauma”[All Fields]) OR “craniocerebral trauma”[All Fields] OR (“head”[All Fields] AND “injury”[All Fields]) OR “head injury”[All Fields])) AND (“phototherapy”[MeSH Terms] OR “phototherapy”[All Fields] OR (“light”[All Fields] AND “therapy”[All Fields]) OR “light therapy”[All Fields] OR ((“light”[MeSH Terms] OR “light”[All Fields] OR “lighted”[All Fields] OR “lights”[All Fields] OR “lighting”[MeSH Terms] OR “lighting”[All Fields] OR “lightings”[All Fields] OR “lightness”[All Fields] OR “lightnesses”[All Fields]) AND (“therapeutics”[MeSH Terms] OR “therapeutics”[All Fields] OR “treatments”[All Fields] OR “therapy”[MeSH Subheading] OR “therapy”[All Fields] OR “treatment”[All Fields] OR “treatment s”[All Fields])) OR (“phototherapy”[MeSH Terms] OR “phototherapy”[All Fields] OR “phototherapies”[All Fields])) AND (“sleep”[MeSH Terms] OR “sleep”[All Fields] OR “sleeping”[All Fields] OR “sleeps”[All Fields] OR “sleep s”[All Fields] OR (“affect”[MeSH Terms] OR “affect”[All Fields] OR “mood”[All Fields]) OR “depress*”[All Fields] OR (“fatiguability”[All Fields] OR “fatiguable”[All Fields] OR “fatigue”[MeSH Terms] OR “fatigue”[All Fields] OR “fatigued”[All Fields] OR “fatigues”[All Fields] OR “fatiguing”[All Fields] OR “fatigueability”[All Fields]) OR (“exhausted”[All Fields] OR “exhausting”[All Fields] OR “exhaustion”[All Fields] OR “exhaustions”[All Fields] OR “exhaustive”[All Fields] OR “exhaustively”[All Fields]) OR (“fatigue”[MeSH Terms] OR “fatigue”[All Fields] OR “tiredness”[All Fields]) OR (“lethargy”[MeSH Terms] OR “lethargy”[All Fields])) AND “random*”[All Fields]	42

4	random*	“random*”[All Fields]	1,553,012
3	sleep OR mood OR depress* OR fatigue OR exhaustion OR tiredness OR lethargy	“sleep”[MeSH Terms] OR “sleep”[All Fields] OR “sleeping”[All Fields] OR “sleeps”[All Fields] OR “sleep s”[All Fields] OR “affect”[MeSH Terms] OR “affect”[All Fields] OR “mood”[All Fields] OR “depress*”[All Fields] OR “fatiguability”[All Fields] OR “fatiguable”[All Fields] OR “fatigue”[MeSH Terms] OR “fatigue”[All Fields] OR “fatigued”[All Fields] OR “fatigues”[All Fields] OR “fatiguing”[All Fields] OR “fatigueability”[All Fields] OR “exhausted”[All Fields] OR “exhausting”[All Fields] OR “exhaustion”[All Fields] OR “exhaustions”[All Fields] OR “exhaustive”[All Fields] OR “exhaustively”[All Fields] OR “fatigue”[MeSH Terms] OR “fatigue”[All Fields] OR “tiredness”[All Fields] OR “lethargy”[MeSH Terms] OR “lethargy”[All Fields]	1,755,558
2	(light therapy) OR (light treatment) OR phototherapy	“phototherapy”[MeSH Terms] OR “phototherapy”[All Fields] OR (“light”[All Fields] AND “therapy”[All Fields]) OR “light therapy”[All Fields] OR ((“light”[MeSH Terms] OR “light”[All Fields] OR “lighted”[All Fields] OR “lights”[All Fields] OR “lighting”[MeSH Terms] OR “lighting”[All Fields] OR “lightings”[All Fields] OR “lightness”[All Fields] OR “lightnesses”[All Fields]) AND (“therapeutics”[MeSH Terms] OR “therapeutics”[All Fields] OR “treatments”[All Fields] OR “therapy”[MeSH Subheading] OR “therapy”[All Fields] OR “treatment”[All Fields] OR “treatment s”[All Fields])) OR (“phototherapy”[MeSH Terms] OR “phototherapy”[All Fields] OR “phototherapies”[All Fields])	280,760
1	(brain injury) OR (head injury)	“brain injuries”[MeSH Terms] OR (“brain”[All Fields] AND “injuries”[All Fields]) OR “brain injuries”[All Fields] OR (“brain”[All Fields] AND “injury”[All Fields]) OR “brain injury”[All Fields] OR (“craniocerebral trauma”[MeSH Terms] OR (“craniocerebral”[All Fields] AND “trauma”[All Fields]) OR “craniocerebral trauma”[All Fields] OR (“head”[All Fields] AND “injury”[All Fields]) OR “head injury”[All Fields])	289,727

B. EMBASE (n = 93)

No.	Query	Results
**#5**	**#1** AND **#2** AND **#3** AND **#4**	**93**
**#4**	**random***	**2,023,880**
**#3**	**sleep** OR **mood** OR **depress*** OR **fatigue** OR **exhaustion** OR **tiredness** OR **lethargy**	**1,597,244**
**#2**	**light** AND **therapy** OR (**light** AND **treatment**) OR **phototherapy**	**244,757**
**#1**	(**'brain'**/exp OR **brain**) AND (**'injury'**/exp OR **injury**) OR ((**'head'**/exp OR **head**) AND (**'injury'**/exp OR **injury**))	**592,766**

C. CINAHL (n = 13)

#	Query	Limiters/Expanders	Last Run Via	Results
S5	S1 AND S2 AND S3 AND S4	Expanders – Apply equivalent subjects<br>Search modes – Find all my search terms	Interface – EBSCOhost Research Databases<br>Search Screen – Advanced Search<br>Database – CINAHL Complete	13
S4	random*	Expanders – Apply equivalent subjects<br>Search modes – Find all my search terms	Interface – EBSCOhost Research Databases<br>Search Screen – Advanced Search<br>Database – CINAHL Complete	479,581
S3	sleep OR mood OR depress* OR fatigue OR exhaustion OR tiredness OR lethargy	Expanders – Apply equivalent subjects<br>Search modes – Find all my search terms	Interface – EBSCOhost Research Databases<br>Search Screen – Advanced Search<br>Database – CINAHL Complete	359,732
S2	(light therapy) OR (light treatment) OR phototherapy	Expanders – Apply equivalent subjects<br>Search modes – Find all my search terms	Interface – EBSCOhost Research Databases<br>Search Screen – Advanced Search<br>Database – CINAHL Complete	23,855
S1	(brain injury) OR (head injury)	Expanders – Apply equivalent subjects<br>Search modes – Find all my search terms	Interface – EBSCOhost Research Databases<br>Search Screen – Advanced Search<br>Database – CINAHL Complete	62,706

D. COCHRANE CENTRAL REGISTER OF CONTROLLED TRIALS (n = 75)
sleep OR mood OR (depress*) OR (fatigue OR exhaustion OR tiredness OR lethargy):ti,ab,kw AND (brain injury) OR (head injury):ti,ab,kw AND (light therapy) OR (light treatment) OR (phototherapy):ti,ab,kw (Word variations have been searched)

S2 Table: Excluded Trials with Reasons

**Trials**	**Reason for exclusion**
Participant mismatch	
O'Brien, et al. 2017[1]	13 participants with stroke and 3 participants with TBI
Intervention mismatch	
Bogdanova, et al. 2017[2]	Transcranial LED helmets and intranasal LED applicators
NCT01725750[3]	Full-spectrum bright light therapy
Non-controlled trials	
Elliott, et al. 2019[4]	Single-arm intervention trial
Trial protocols without results	
NCT01175993[5]	Effects of Rapid-Resisted Exercise and Bright Light Therapy on Ambulatory Adults With Traumatic Brain Injury
NCT03578003[6]	Morning Bright Light to Improve Sleep Quality in Veterans
NCT03785600[7]	Improving Sleep in Veterans With TBI
NCT03968874[8]	Improving Sleep, Sleep-related Outcomes, and Biomarkers in Veterans

References

1. O'Brien, K. *et al.* Randomized trial of light therapy for cognitive function during inpatient rehabilitation for acquired brain injury. *Archives of Physical Medicine and Rehabilitation* **98**, e173–e174 (2017).
2. Bogdanova, Y. *et al.* Transcranial LED treatment for cognitive dysfunction and sleep in chronic TBI: Randomized controlled pilot trial. *Archives of Physical Medicine and Rehabilitation* **98**, e122–e123 (2017).
3. NCT01725750. Treatment of Post-Traumatic Brain Injury (Post-TBI) Fatigue With Light Therapy. *https://clinicaltrials.gov/show/NCT01725750* (2012).
4. Elliott, J. E., Balba, N. M., McBride, A. A., Thomas, S. V. & Lim, M. M. Morning bright light improves insomnia, mood, and pain in veterans with TBI and PTSD. *Sleep* **42**, A349 (2019).
5. NCT01175993. Effects of Rapid-Resisted Exercise and Bright Light Therapy on Ambulatory Adults With Traumatic Brain Injury. *https://clinicaltrials.gov/show/NCT01175993* (2010).
6. NCT03578003. Morning Bright Light to Improve Sleep Quality in Veterans. *https://clinicaltrials.gov/show/NCT03578003* (2018).
7. NCT03785600. Improving Sleep in Veterans With TBI. *https://clinicaltrials.gov/show/NCT03785600* (2018).
8. NCT03968874. Improving Sleep, Sleep-related Outcomes, and Biomarkers in Veterans. *https://clinicaltrials.gov/show/NCT03968874* (2019).

SUPPLEMENTARY FIGURES

S1 Fig.: Forest plots comparing the dropout rates [blue-wavelength light therapy (BWLT) vs. control interventions (amber, red, or no light therapy)].

	BWLT		Control			Risk Ratio
**Study**	**Events**	**Total**	**Events**	**Total**	**Weight**	**MH, Random, 95% CI**
Sinclair 2014	0	10	0	20	3.3%	1.95 [0.04; 91.63]
Quera Salva 2019	0	10	0	10	3.3%	1.00 [0.02; 45.85]
Killgore 2020	2	16	4	16	20.2%	0.50 [0.11; 2.35]
Raikes 2020	3	17	1	18	10.3%	3.18 [0.36; 27.65]
Bell 2021	6	65	9	66	51.0%	0.68 [0.26; 1.79]
Connolly 2021	1	17	5	13	11.9%	0.15 [0.02; 1.16]
**Total (95% CI)**		**135**		**143**	**100.0%**	**0.66 [0.33; 1.32]**

Heterogeneity: $Tau^2 = 0$; $Chi^2 = 4.51$, df = 5 (P = 0.48); $I^2 = 0\%$
Test for overall effect: Z = -1.18 (P = 0.24)

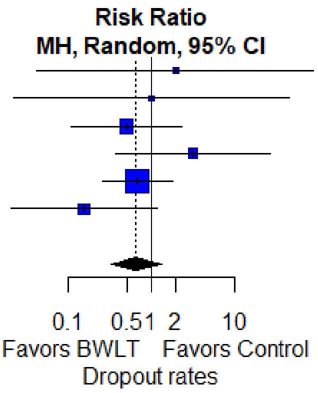

S2 Fig.: Subgroup analysis of five outcomes - based on the severity of traumatic brain injury.

Study or Subgroup	BWLT Mean	BWLT SD	BWLT Total	Control Mean	Control SD	Control Total	Weight	Std. Mean Difference IV, Random, 95% CI
**Severity Level = More than mild**								
Sinclair 2014	-4.70	1.66	10	-2.10	1.36	20	16.3%	-1.73 [-2.62; -0.84]
Quera Salva 2019	-1.70	2.58	10	-2.30	5.06	10	16.3%	0.14 [-0.73; 1.02]
Bell 2021	-0.40	2.29	62	0.00	2.29	59	18.6%	-0.17 [-0.53; 0.18]
Connolly 2021	-1.00	0.87	16	-1.26	1.01	8	16.5%	0.27 [-0.58; 1.13]
**Total (95% CI)**			**98**			**97**	**67.7%**	**-0.35 [-1.21; 0.50]**
Heterogeneity: $Tau^2 = 0.60$; $Chi^2 = 12.96$, df = 3 ($P < 0.01$); $I^2 = 77\%$								
**Severity Level = Mild**								
Killgore 2020	-25.35	19.42	14	8.84	19.31	12	16.1%	-1.71 [-2.63; -0.79]
Raikes 2020	-1.35	0.62	17	0.61	0.90	18	16.2%	-2.46 [-3.37; -1.56]
**Total (95% CI)**			**31**			**30**	**32.3%**	**-2.09 [-2.83; -1.35]**
Heterogeneity: $Tau^2 = 0.07$; $Chi^2 = 1.32$, df = 1 ($P = 0.25$); $I^2 = 24\%$								
**Total (95% CI)**			**129**			**127**	**100.0%**	**-0.92 [-1.84; 0.00]**
Heterogeneity: $Tau^2 = 1.15$; $Chi^2 = 40.38$, df = 5 ($P < 0.01$); $I^2 = 88\%$								
Test for overall effect: $Z = -1.95$ ($P = 0.05$)								
Test for subgroup differences: $Chi^2 = 9.13$, df = 1 ($P < 0.01$)								

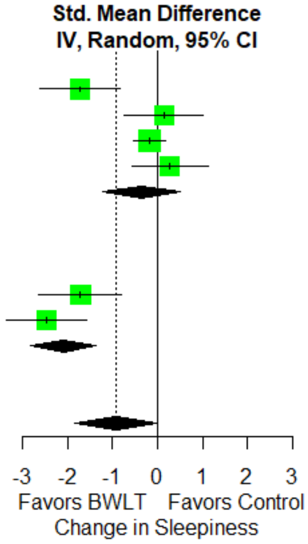

A)

Study or Subgroup	BWLT Mean	BWLT SD	BWLT Total	Control Mean	Control SD	Control Total	Weight	Std. Mean Difference IV, Random, 95% CI
**Severity Level = More than mild**								
Sinclair 2014	-1.40	0.67	10	-0.85	0.93	20	20.8%	-0.63 [-1.40; 0.15]
Quera Salva 2019	-27.76	37.56	10	24.33	62.10	10	17.8%	-0.97 [-1.91; -0.03]
Connolly 2021	-0.13	0.82	16	0.16	0.58	8	19.3%	-0.37 [-1.23; 0.48]
**Total (95% CI)**			**36**			**38**	**57.9%**	**-0.64 [-1.13; -0.15]**
Heterogeneity: $Tau^2 = 0$; $Chi^2 = 0.86$, df = 2 ($P = 0.65$); $I^2 = 0\%$								
**Severity Level = Mild**								
Killgore 2020	-1.71	0.15	14	-2.00	1.63	12	20.9%	0.25 [-0.52; 1.03]
Raikes 2020	-1.30	0.08	17	-0.73	0.53	18	21.3%	-1.45 [-2.20; -0.69]
**Total (95% CI)**			**31**			**30**	**42.1%**	**-0.60 [-2.27; 1.07]**
Heterogeneity: $Tau^2 = 1.30$; $Chi^2 = 9.52$, df = 1 ($P < 0.01$); $I^2 = 89\%$								
**Total (95% CI)**			**67**			**68**	**100.0%**	**-0.63 [-1.21; -0.05]**
Heterogeneity: $Tau^2 = 0.27$; $Chi^2 = 10.38$, df = 4 ($P = 0.03$); $I^2 = 61\%$								
Test for overall effect: $Z = -2.12$ ($P = 0.03$)								
Test for subgroup differences: $Chi^2 = 0.00$, df = 1 ($P = 0.97$)								

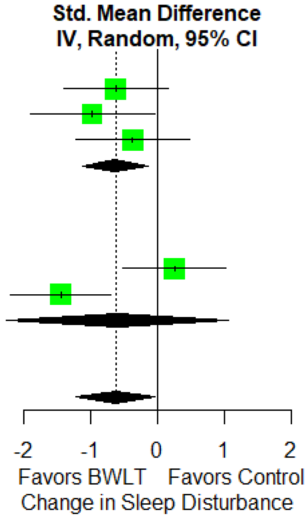

B)

Study or Subgroup	BWLT Mean	BWLT SD	BWLT Total	Control Mean	Control SD	Control Total	Weight	Std. Mean Difference IV, Random, 95% CI
**Severity Level = More than mild**								
Sinclair 2014	-6.10	1.56	10	-1.55	3.39	20	24.1%	-1.51 [-2.37; -0.65]
Quera Salva 2019	-1.69	2.49	10	0.80	2.53	10	22.2%	-0.95 [-1.89; -0.01]
**Total (95% CI)**			**20**			**30**	**46.3%**	**-1.25 [-1.89; -0.62]**
Heterogeneity: $Tau^2 = 0$; $Chi^2 = 0.75$, df = 1 ($P = 0.39$); $I^2 = 0\%$								
**Severity Level = Mild**								
Killgore 2020	4.00	5.07	14	4.83	3.88	12	26.5%	-0.18 [-0.95; 0.60]
Raikes 2020	-2.47	1.12	17	0.39	2.55	18	27.2%	-1.40 [-2.15; -0.66]
**Total (95% CI)**			**31**			**30**	**53.7%**	**-0.79 [-2.00; 0.41]**
Heterogeneity: $Tau^2 = 0.60$; $Chi^2 = 5.01$, df = 1 ($P = 0.03$); $I^2 = 80\%$								
**Total (95% CI)**			**51**			**60**	**100.0%**	**-1.00 [-1.62; -0.38]**
Heterogeneity: $Tau^2 = 0.22$; $Chi^2 = 6.85$, df = 3 ($P = 0.08$); $I^2 = 56\%$								
Test for overall effect: $Z = -3.17$ ($P < 0.01$)								
Test for subgroup differences: $Chi^2 = 0.44$, df = 1 ($P = 0.51$)								

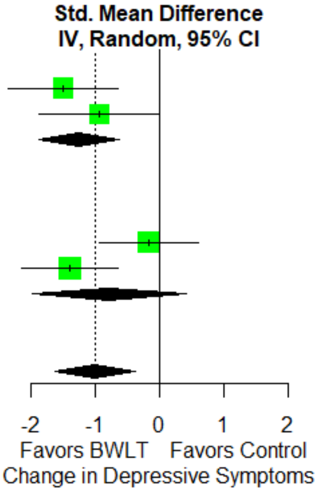

C)

Study or Subgroup	BWLT Mean	BWLT SD	BWLT Total	Control Mean	Control SD	Control Total	Weight	Std. Mean Difference IV, Random, 95% CI
**Severity Level = More than mild**								
Sinclair 2014	-1.30	0.38	10	-0.15	0.24	20	23.0%	-3.83 [-5.11; -2.54]
Quera Salva 2019	-1.18	1.43	10	0.29	1.17	10	24.8%	-1.08 [-2.03; -0.13]
Bell 2021	-10.20	15.70	62	-8.10	15.70	59	27.1%	-0.13 [-0.49; 0.22]
Connolly 2021	-0.71	0.55	16	-0.12	0.59	8	25.1%	-1.01 [-1.92; -0.11]
**Total (95% CI)**			**98**			**97**	**100.0%**	**-1.44 [-2.95; 0.08]**
Heterogeneity: Tau^2 = 2.16; Chi^2 = 32.46, df = 3 (P < 0.01); I^2 = 91%								
**Total (95% CI)**			**98**			**97**	**100.0%**	**-1.44 [-2.95; 0.08]**

Heterogeneity: Tau^2 = 2.16; Chi^2 = 32.46, df = 3 (P < 0.01); I^2 = 91%
Test for overall effect: Z = -1.86 (P = 0.06)
Test for subgroup differences: Chi^2 = 0.00, df = 0 (P = NA)

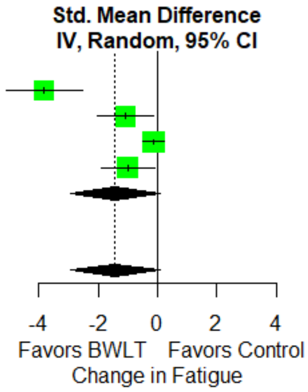

D)

Study or Subgroup	BWLT Events	BWLT Total	Control Events	Control Total	Weight	Risk Ratio MH, Random, 95% CI
**Severity Level = More than mild**						
Sinclair 2014	0	10	0	20	3.3%	1.95 [0.04; 91.63]
Quera Salva 2019	0	10	0	10	3.3%	1.00 [0.02; 45.85]
Bell 2021	6	65	9	66	51.0%	0.68 [0.26; 1.79]
Connolly 2021	1	17	5	13	11.9%	0.15 [0.02; 1.16]
**Total (95% CI)**		**102**		**109**	**69.5%**	**0.56 [0.24; 1.30]**
Heterogeneity: Tau^2 = 0; Chi^2 = 2.22, df = 3 (P = 0.53); I^2 = 0%						
**Severity Level = Mild**						
Killgore 2020	2	16	4	16	20.2%	0.50 [0.11; 2.35]
Raikes 2020	3	17	1	18	10.3%	3.18 [0.36; 27.65]
**Total (95% CI)**		**33**		**34**	**30.5%**	**1.07 [0.18; 6.39]**
Heterogeneity: Tau^2 = 0.79; Chi^2 = 1.85, df = 1 (P = 0.17); I^2 = 46%						
**Total (95% CI)**		**135**		**143**	**100.0%**	**0.66 [0.33; 1.32]**

Heterogeneity: Tau^2 = 0; Chi^2 = 4.51, df = 5 (P = 0.48); I^2 = 0%
Test for overall effect: Z = -1.18 (P = 0.24)
Test for subgroup differences: Chi^2 = 0.41, df = 1 (P = 0.52)

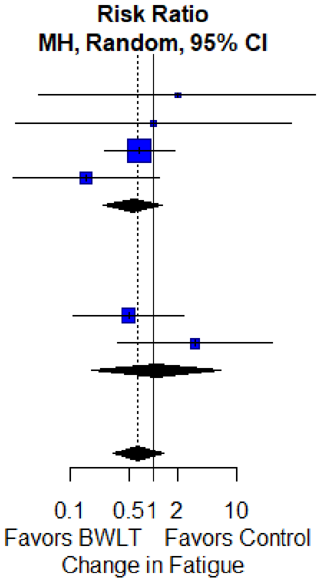

E)

S3 Fig.: Sensitivity analysis of five outcomes – excluding three trials with a high-risk of bias.

Study	BWLT Mean	BWLT SD	BWLT Total	Control Mean	Control SD	Control Total	Weight	Std. Mean Difference IV, Random, 95% CI
Killgore 2020	-25.35	19.42	14	8.84	19.31	12	31.8%	-1.71 [-2.63; -0.79]
Raikes 2020	-1.35	0.62	17	0.61	0.90	18	32.0%	-2.46 [-3.37; -1.56]
Bell 2021	-0.40	2.29	62	0.00	2.29	59	36.2%	-0.17 [-0.53; 0.18]
**Total (95% CI)**			**93**			**89**	**100.0%**	**-1.39 [-2.76; -0.03]**

Heterogeneity: $Tau^2 = 1.31$; $Chi^2 = 27.47$, df = 2 ($P < 0.01$); $I^2 = 93\%$
Test for overall effect: Z = -2.00 (P = 0.05)

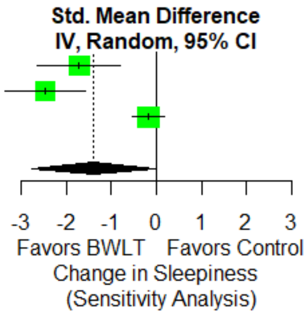

A)

Study	BWLT Mean	BWLT SD	BWLT Total	Control Mean	Control SD	Control Total	Weight	Std. Mean Difference IV, Random, 95% CI
Killgore 2020	-1.71	0.15	14	-2.00	1.63	12	49.9%	0.25 [-0.52; 1.03]
Raikes 2020	-1.30	0.08	17	-0.73	0.53	18	50.1%	-1.45 [-2.20; -0.69]
**Total (95% CI)**			**31**			**30**	**100.0%**	**-0.60 [-2.27; 1.07]**

Heterogeneity: $Tau^2 = 1.30$; $Chi^2 = 9.52$, df = 1 ($P < 0.01$); $I^2 = 89\%$
Test for overall effect: Z = -0.71 (P = 0.48)

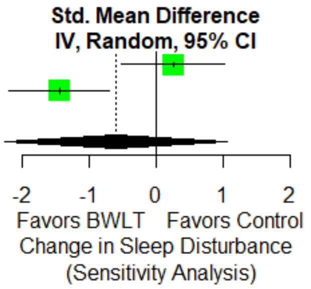

B)

Study	BWLT Mean	BWLT SD	BWLT Total	Control Mean	Control SD	Control Total	Weight	Std. Mean Difference IV, Random, 95% CI
Killgore 2020	4.00	5.07	14	4.83	3.88	12	49.7%	-0.18 [-0.95; 0.60]
Raikes 2020	-2.47	1.12	17	0.39	2.55	18	50.3%	-1.40 [-2.15; -0.66]
**Total (95% CI)**			**31**			**30**	**100.0%**	**-0.79 [-2.00; 0.41]**

Heterogeneity: $Tau^2 = 0.60$; $Chi^2 = 5.01$, df = 1 ($P = 0.03$); $I^2 = 80\%$
Test for overall effect: Z = -1.29 (P = 0.20)

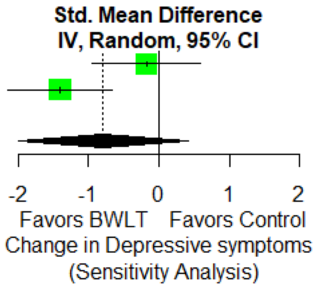

C)

Study	BWLT Mean	BWLT SD	BWLT Total	Control Mean	Control SD	Control Total	Std. Mean Difference IV, 95% CI
Bell 2021	-10.20	15.70	62	-8.10	15.70	59	-0.13 [-0.49; 0.22]

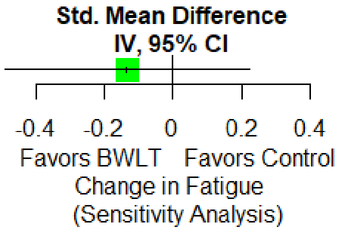

D)

Study	BWLT Events	BWLT Total	Control Events	Control Total	Weight	Risk Ratio MH, Random, 95% CI
Killgore 2020	2	16	4	16	24.7%	0.50 [0.11; 2.35]
Raikes 2020	3	17	1	18	12.7%	3.18 [0.36; 27.65]
Bell 2021	6	65	9	66	62.6%	0.68 [0.26; 1.79]
**Total (95% CI)**		**98**		**100**	**100.0%**	**0.76 [0.35; 1.65]**

Heterogeneity: $Tau^2 < 0.01$; $Chi^2 = 2.01$, df = 2 (P = 0.37); $I^2 = 1\%$
Test for overall effect: Z = -0.68 (P = 0.49)

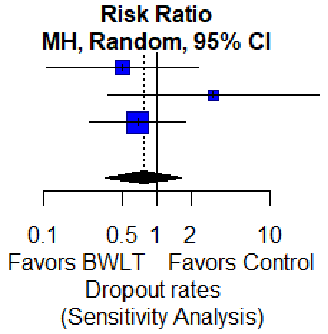

E)

S4 Fig.: Funnel plot showing the treatment-effect estimates of dropout rates.

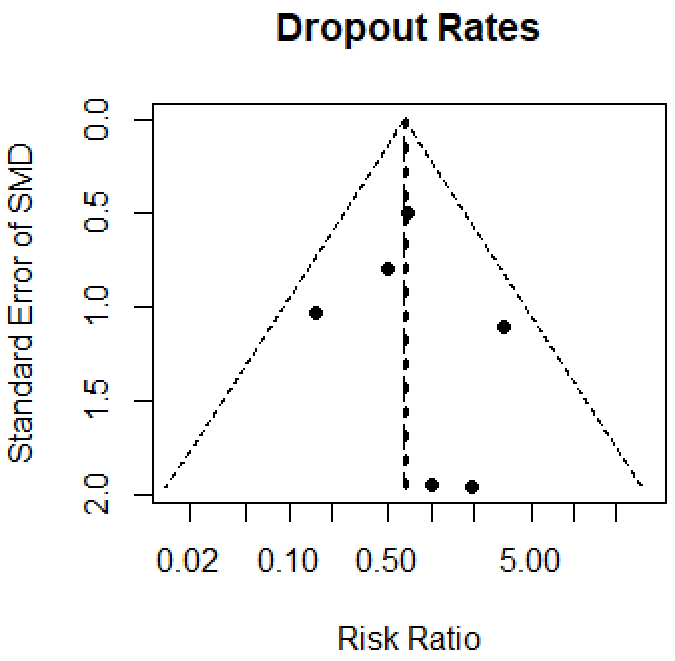

Supplement: S1 Appendix — (PDF) [file pone.0274025.s002.pdf]
