# Supplementary material for: Efficacy and acceptability of blue-wavelength light therapy for post-TBI behavioral symptoms: A systematic review and meta-analysis of randomized controlled trials
Source: PLoS One. 2022 Oct 6;17(10):e0274025. doi: 10.1371/journal.pone.0274025 (PMC9536631; doi:10.1371/journal.pone.0274025)
Supplement: Attachment — Submitted filename: Response to Reviewer.docx [file pone.0274025.s003.docx]

Response to Reviewer’s Comments

Thank you for the valued comments given by the reviewer. They are very much helpful in improving our manuscript. We have revised the manuscript accordingly and wish to give details on each point of revision in the following statements. In the mark-up copy, the revision points are in red. The pages and paragraphs are also referred to those in the mark-up copy of the manuscript. Please be informed that the cleaned manuscript is for press and has no mark-up inside.

1. The authors have mentioned a very detailed inclusion criteria. However, the manuscript lacks any statement regarding the exclusion criteria

- Our response: We have added the following sentence.
- Page 5, Paragraph 1: To maintain the data consistency, we excluded the following trials: i) less than 50% of the participants having TBI, ii) light therapy not perceived by the eyes, and iii) concomitant treatment of biological or physical therapy.

2. More information should be mentioned about WebPlotDigitizer. Moreover, adding a reference would be ideal.

- Our response: We have added the version and reference #24 of the WebPlotDigitizer.
- Page 6, Paragraph 3: We extracted the data from published articles and,…WebPlotDigitizer, version 4.5 [24].
- Reference #24: Rohatgi A. WebPlotDigitizer. Pacifica, California, USA; 2011. Available: <https://automeris.io/WebPlotDigitizer>

3. Can the authors add the company names of the software used in the meta-analysis ?

- Our response: We have added the company names of the software used in the meta-analysis.
- Page 8, Paragraph 3: All analyses were performed in the R Program (version 4.2), R Foundation for Statistical Computing [36].

4. It is necessary to cite which trials reported administering BWLT via light box or glass or house lighting.

- Our response: We have added citations after each device.
- Page 9, Last paragraph & Page 10, Paragraph 1: Devices used to administer light therapy were lightboxes (4 trials) [22,26,39,41], face-mounted device resembling glasses (1 trial) [40], and house lighting (1 trial) [38].

5. In my opinion, the authors should emphasize on the strengths of this article. The authors need to highlight what are the gaps in the literature that required this study, and that how the results of this review help with filling the gaps in the medical literature.

- Our response: We have added a paragraph to emphasize the strength of this article.
- Page 20, Paragraph 2: This meta-analysis included the largest number of RCTs examining the benefits of BWLT for post-TBI behavioral symptoms. While the previous results were mixed and inconclusive, the results of this meta-analysis seemed to clarify at least two points: i) BWLT had a large treatment effect in reducing depressive symptoms (moderate certainty), and ii) BWLT had a moderate traetment effect in relieving sleep disturbance (low certainty). These results should be helpful for research and treatment planning in this area.

6. Regarding the "Table 1" :-

Overall, the manuscript is very well written and I appreciate that the authors have presented accurate results. However, please note that the table in its current form is very confusing. The table should have proper sections for control/intervention and sample/age/bmi/duration/female or male percentage/characteristics/outcomes.

- Our response: We have extensively revised Table 1 accordingly to the reviewer’s comment
- Page 11-12: Please see Table 1.

**Remark**

Please be informed that we have added three sentences to inform about the trials with two and three arms that included in this study

Page 9, Paragraph 3: Of the six trials, one had three arms of BWLT (n = 10), LWLT (ambient light therapy) (n = 10), and NLT (n = 10) [26]. The data of LWLT and NLT groups of this three-arm trial were combined as a control group. The other five trials had two arms comparing BWLT and LWLT/NLT.

Thank you for your consideration.
