# Supplementary material for: Efficacy and acceptability of blue-wavelength light therapy for post-TBI behavioral symptoms: A systematic review and meta-analysis of randomized controlled trials
Source: PLoS One. 2022 Oct 6;17(10):e0274025. doi: 10.1371/journal.pone.0274025 (PMC9536631; doi:10.1371/journal.pone.0274025)
Supplement: Attachment — Submitted filename: Response to Reviewer 20220817.docx [file pone.0274025.s004.docx]

Response to Reviewer’s Comments

Thank you for the valued comments given by the reviewer. They are very much helpful in improving our manuscript. We have further revised the manuscript accordingly and wish to give details on each point of revision in the following statements. In the mark-up copy, the revision points are in red. The pages and paragraphs are also referred to those in the mark-up copy of the manuscript. Please be informed that the cleaned manuscript is for press and has no mark-up inside.

1. Regarding the strengths of the findings, the authors need to improve the paragraph they have added regarding the strengths of the manuscript:

- They can improve by telling how these findings help in clinical practice.

- Further emphasis on how these findings translate into improved prognosis and management of post traumatic brain injury patients.

- Additionally, they may also discuss briefly about how these findings help in improving the current guidelines and recommendations.

Thank you for your consideration.

- Our response: We consider that all reviewer’s comments are relevant to the implications of our study findings for clinical practice and research. We, therefore, add a paragraph on this matter between the parts of limitations and Conclusions of the Discussion. In addition, we add a reference to support two sentences in the added paragraph.
- Page 21, Paragraph 5 – Page 22, Paragraph 1:

The present results should be helpful for clinical practice and research in this area. Because treatment options for post-TBI behavioral symptoms remain unsatisfactory, as a promising intervention, BWLT may change the clinical practice and prognosis of patients with post-TBI depression and sleep disturbance. Clinicians may consider BWLT as a treatment option for post-TBI behavioral symptoms. In a recent review [55], guidelines suggested only neurobehavioral interventions and cognitive-behavioral therapy for rehabilitating behavioral and emotional disorders. The low to moderate certainty of our positive findings may impact future evidence-based practice guidelines and lead to a recommendation of BWLT for some post-TBI behavioral symptoms, e.g., depression, sleep disturbance. More trials in large sample sizes with longer study duration remain needed. Two reasons appear to support that patients with mild TBI should be a priority studied group. First, other than the benefits for depression and sleep disturbance, our subgroup analysis also found that BWLT might improve sleepiness in patients with mild TBI. Second, little has been known about the specific therapy for behavioral symptoms in mild TBI [55].

- Page 30, reference 55:

55. Lee SY, Amatya B, Judson R, Truesdale M, Reinhardt JD, Uddin T, et al. Clinical practice guidelines for rehabilitation in traumatic brain injury: a critical appraisal. Brain Inj. 2019;33: 1263–1271. doi:10.1080/02699052.2019.1641747
